# Relationship between hemoglobin and red blood cell distribution width ratio and albuminuria in the United States adults aged 20 years and above: a cross-sectional analysis

**DOI:** 10.3389/fnut.2025.1572196

**Published:** 2025-11-13

**Authors:** Guangxin Li, Guangchao Li, Wenyu Xu, Yanping Yang, Jin-an Zhang, Jing Zhang

**Affiliations:** 1 Department of Endocrinology & Rheumatology, Shanghai University of Medicine & Health Sciences Affiliated Zhoupu Hospital, Shanghai, China; 2 Graduate School, Heilongjiang University of Chinese Medicine, Harbin, China

**Keywords:** hemoglobin to red cell distribution width ratio, albuminuria, NHANES, cross-sectional study, nonlinear correlation

## Abstract

**Objective:**

Hemoglobin to red blood cell distribution width ratio (HRR) is a novel prognostic indicator of disease, and is also associated with a variety of chronic systemic diseases. However, there are fewer studies on the relationship between HRR and albuminuria. This study aimed to investigate the association between the two subjects.

**Methods:**

A total of 13,971 participants aged 20 and older in the National Health and Nutrition Examination Survey (NHANES) between 2007 and 2018. The HRR is the ratio of hemoglobin to the width of the red blood cell distribution. Smoothed curve fitting and multivariate segmented linear regression models were used to study the relationship between HRR and albuminuria. Finally, subgroup analysis and interaction tests were performed to test the stability of the results.

**Results:**

The results of the univariate analysis showed that HRR was negatively associated with albuminuria (OR = 0.86, *p* < 0.0001). After further adjustment of potential confounding factors, the smooth curve fitting showed that HRR was nonlinearly related to albuminuria. The inflection point of HRR in multiple segmented linear regression analysis was 1.213. Specifically, when HRR <1.213, (OR = 0.93, 95% CI 0.90, 0.97; *p* = 0.0007); when HRR ≥ 1.213, (OR = 1.24, 95% CI 1.08, 1.43; *p* < 0.0001). Subgroup analyses revealed that this association exists as stable in different populations.

**Conclusion:**

There was a nonlinear correlation between HRR and albuminuria in adults aged 20 or older in the United States.

## Introduction

Albuminuria, characterized by abnormal excretion of protein in the urine, it is usually expressed as the ratio of urinary albumin to creatinine in urine (UACR) over 30 mg/g (1, 2). Microalbuminuria is an early stage of glomerular disease, and the presence of albuminuria indicates an impaired glomerular filtration barrier, the first line of defense for the maintenance of the renal selective permeability (3, 4). Previous studies have shown that even low levels of albuminuria are associated with diabetic nephropathy, all-cause death, and cardiovascular death (5, 6). This damage triggers a range of harmful effects over time. Persistent albuminuria was not only associated with increased likelihood of developing end-stage renal disease but also with the occurrence of adverse cardiovascular events (4, 7). Therefore, early detection and effective management of albuminuria is essential to reduce the risk of renal failure and associated complications.

HRR was originally proposed as a new combinatorial biomarker by Sun et al. (8) in the Non-Small Cell Lung Cancer Progression Study. HRR has been widely used to assess various diseases such as cancer (9), cardiovascular disease (10), cerebrovascular disease (11, 12), and bone mineral density (13) etc. HRR, calculated by dividing hemoglobin by red blood cell distribution width ratio (RDW), is a new inflammatory marker that better reflects the function of red blood cells. Given the emerging evidence that inflammation plays a key role in the pathophysiology of albuminuria (14, 15), the potential association between HRR and albuminuria has been noted. HRR is unique in its ability to reflect subtle changes in blood parameters that may not be detected when analyzing hemoglobin or red blood cell distribution width (RDW) alone.

In the assessment and diagnosis of the disease, the complete blood cell count (CBC) plays a key role in revealing the blood health status of individuals (16). Hemoglobin is an important parameter of CBC. It is a protein rich in red blood cells (RBC), which mainly plays the role of oxygen transport molecule (17). Due to the direct link between blood circulation and tissue oxygen supply, the hemoglobin level is closely related with the occurrence and development of albuminuria. However, previous studies on the relationship between hemoglobin levels and albuminuria have drawn inconsistent conclusions. A retrospective cohort study in Japan found that low hemoglobin concentration was associated with the progression and development of albuminuria in patients with type 2 diabetes (18). However, another study showed that patients with erycytosis (female, hemoglobin 190 g / L or above; male, 210 g/L or above) had a median urinary protein level of 2.5 g/24 h, and the kidney is one of the most severely affected organs (19). Furthermore, a cross-sectional study with a total population of 8,868 found that, after adjusting for all latent variables, hemoglobin levels showed a U-type association with albuminuria, with an inflection point of 15.5 g/dL, both lower and higher hemoglobin levels were associated with the development of albuminuria (20). RDW is another important parameter of CBC and is used to measure variability in RBC counts. Existing studies found a significant negative relationship between RDW and renal function, and this association remained highly significant even after multivariable adjustment for comorbidities, iron deficiency, inflammation and nutritional status (21). HRR is a ratio that combines hemoglobin and RDW, and there is a lack of clarity regarding the relationship between HRR and albuminuria; therefore, we conducted the present study to examine the relationship between HRR and albuminuria in the United States adults.

## Materials and methods

### Study population

**Figure 1:**
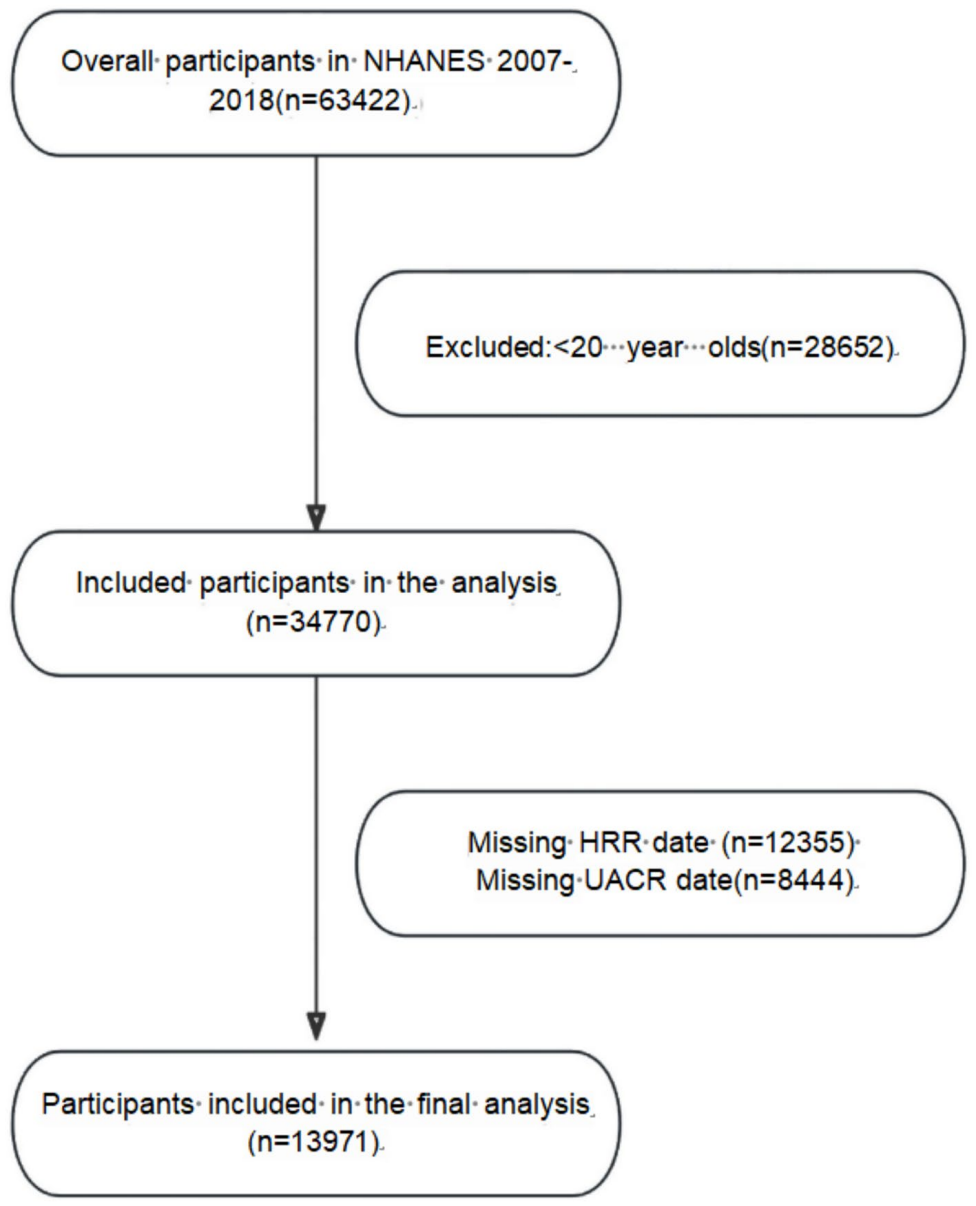
Flow chart of the participants.

Data for this study were obtained from the National Health and Nutrition Examination Survey of the National Center for Health Statistics (NHANES), the project was approved by the Ethics Review Board of the National Center for Health Statistics (NCHS), and all participants signed written informed consent before enrollment. This study covers the data from 2007 to 2018, involving a total of 63,422 subjects. During the screening process, we excluded participants under 20 years (28652), participants with incomplete urinary albumin creatinine ratio (UACR) data (8444) and participants with incomplete HRR data (12355). A total of 13,971 individuals were ultimately included in the study. The specific screening process is shown in Figure 1.

### Exposure variables and outcome definition

The exposure variable in this study was HRR. The HRR value of each subject was calculated by dividing hemoglobin (unit: grams/deciliter, g/dL) by RDW, and the results were rounded to two decimal places. Blood samples from NHANES participants were collected at standardized flow sampling centers. The NHANES Laboratory/Medical Technician Operation Manual details the specific procedures for sample collection and processing. CBC parameters including hemoglobin and RDW were derived using the Beckman Coulter methodology.

The definition of proteinuria is based on the urinary albumin to creatinine ratio (UACR), which is calculated by dividing the concentration of urinary albumin (mg) by the concentration of urinary creatinine (g). UACR> 30 mg/g is defined as albuminuria (2). The researchers collected urine samples from NHANES participants at standardized mobile testing centers. Urinary albumin levels were measured using solid-phase fluorescence immunoassay, while urinary creatinine levels were assessed using enzymatic methods.

### Covariates

Based on previous studies (22), this research included the following covariates: age, gender, race, poverty-income ratio (PIR), education level, body mass index (BMI), smoking and alcohol consumption status, blood urea nitrogen (BUN), serum creatinine (CR), uric acid (UA), estimated glomerular filtration rate (eGFR), alanine aminotransferase (ALT), aspartate aminotransferase (AST), low-density lipoprotein cholesterol (LDL-C), high-density lipoprotein cholesterol (HDL-C), triglycerides (TG), total cholesterol (TC), fasting blood glucose (FBG), hemoglobin A1c (HbA1c), as well as hypertension, diabetes, and cardiovascular disease (CVD).

Demographic data were collected through questionnaires. The specific items included: age (years); gender (male or female); ethnicity (Mexican American, other Hispanic, non-Hispanic White, non-Hispanic Black, and other ethnic groups); education level (less than high school, high school, more than high school); and poverty income ratio (PIR).

The physical examination included measurements of height, weight, systolic blood pressure (SBP), and diastolic blood pressure (DBP). BMI was calculated based on height (HT, meters) and weight (WT, kilograms) using the formula BMI = WT/(HT)^2.

Laboratory data included BUN, CR, UA, ALT, AST, LDL-C, HDL-C, TG, TC, FBG, HbA1c and eGFR, among which eGFR was calculated by CKD-EPI formula (23).

We collected participants’ personal behaviors, medical history, and medication use through questionnaires. The criteria included: (1) Those who have smoked at least 100 cigarettes in their lifetime are classified as smokers; (2) Those who have consumed at least 12 alcoholic beverages in the past 12 months are classified as drinkers; (3) Hypertension was defined as three consecutive measurements showing an average SBP ≥ 140 mmHg and/or DBP ≥ 90 mmHg, or two or more participants being diagnosed with hypertension or prescribed antihypertensive medications; (4) Diabetes was defined as being diagnosed by a physician, taking anti-diabetic medications, using insulin, or having FBG ≥ 7.0 mmol/LmM or HbA1c ≥ 6.5%; (5) CVD were self-reported conditions including coronary heart disease, angina pectoris, heart failure, myocardial infarction, and/or stroke.

### Statistical analysis

If the continuous variables meet normal distribution, expressed as mean ± standard deviation; If the continuous variable shows a skewed distribution, expressed as the median (Q1-Q3), Categorical variables were expressed as percentages. First, the linear relationship between HRR and albuminuria was analyzed using univariate logistic regression, and then, after adjusting for potential confounders, the nonlinear relationship between HRR and albuminuria was investigated using smoothed curve fitting and multivariate segmented linear regression models. Finally, subgroup analysis and interaction tests were performed to determine the stability of the relationship between HRR and albuminuria in different populations by age (≥60, <60 years), gender (male, female), BMI (≥25, <25), eGFR (≥60, <60), race, diabetes, hypertension, cardiovascular disease, smoking and drinking status. The missing covariates were reanalyzed after multiple interpolation, and multivariate logistic regression analysis was performed to ensure the stability of the research results (24, 25). A value of *p* < 0.05 was considered statistically significant. All data were analyzed using the statistical packages R (2The R Foundation) and EmpowerStats (3X&Y Solutions, Inc., Boston, MA, USA conduct).

## Results

### Subject population description

Table 1 describes the baseline characteristics of the researchers. A total of 13,971 investigators were included in this study, of whom 6,931 (49.61%) were males and 7,074 (50.39%) were females. The mean age was 49.29 ± 17.66 years; mean HRR was 1.07 ± 0.17 and overall prevalence of albuminuria was 12.1%. Among the study population, 35.94% were patients with hypertension, 18.50% were patients with diabetes and 8.16% were patients with CVD.

**Table 1:** Study population description.

Variables	Total (*n* = 13,971)	No-Albuminuria (*n* = 11,953)	Albuminuria (*n* = 2018)	*p* value
Age (years)	49.29 ± 17.66	47.74 ± 17.25	58.43 ± 17.33	<0.001
BMI (kg/m^2^)	29.25 ± 6.87	29.17 ± 6.80	29.72 ± 7.26	<0.001
PIR	2.48 ± 1.62	2.51 ± 1.63	2.26 ± 1.54	<0.001
HDL-C (mg/dL)	53.00 ± 16.32	53.07 ± 16.06	52.57 ± 17.79	0.199
LDL-C (mmol/L)	2.82 ± 1.02	2.87 ± 0.99	2.53 ± 1.15	<0.001
TC (mmol/L)	4.63 ± 1.55	4.75 ± 1.42	3.91 ± 2.04	<0.001
TG (mg/dL)	121.00 (81.00–189.00)	120.00 (79.00–186.00)	134.00 (88.00–210.00)	<0.001
ALT (u/L)	21.00 (16.00–28.00)	21.00 (16.00–28.00)	20.00 (16.00–28.00)	0.118
AST(u/L)	26.12 ± 17.66	25.83 ± 16.27	27.88 ± 24.24	<0.001
FBG (mmol/L)	5.72 ± 2.23	5.54 ± 1.88	6.74 ± 3.51	<0.001
HbA1c(%)	5.78 ± 1.10	5.69 ± 0.93	6.34 ± 1.70	<0.001
BUN (mg/dL)	13.61 ± 5.87	13.15 ± 5.03	16.34 ± 8.95	<0.001
UA (mg/dL)	5.46 ± 1.44	5.41 ± 1.41	5.79 ± 1.60	<0.001
CR (mg/dL)	0.90 ± 0.39	0.87 ± 0.24	1.07 ± 0.82	<0.001
eGFR (mL/min/1.73 m^2^)	92.55 ± 26.67	94.31 ± 25.32	82.16 ± 31.63	<0.001
HRR	1.07 ± 0.17	1.08 ± 0.16	1.04 ± 0.18	<0.001
Sex [*N* (%)]				0.635
Male	6,931 (49.61%)	5,920 (49.53%)	1,011 (50.10%)	
Female	7,074 (50.39%)	6,033 (50.47%)	1,007 (49.90%)	
Race [*N* (%)]				0.006
Mexican American	2,128 (15.23%)	1824 (15.26%)	304 (15.06%)	
Other Hispanic	1,485 (10.63%)	1,287 (10.77%)	198 (9.81%)	
Non-Hispanic White	6,069 (43.44%)	5,157 (43.14%)	912 (45.19%)	
Non-Hispanic Black	2,828 (20.24%)	2,394 (20.03%)	434 (21.51%)	
Other race	1,461 (10.46%)	1,291 (10.80%)	170 (8.42%)	
Smoking [*N* (%)]				<0.001
No	7,689 (55.04%)	6,674 (55.84%)	1,015 (50.30%)	
Yes	6,282 (44.96%)	5,279 (44.16%)	1,003 (49.70%)	
Drinking [*N* (%)]				<0.001
No	3,868 (27.69%)	3,228 (27.01%)	640 (31.71%)	
Yes	10,103 (72.31%)	8,725 (72.99%)	1,378 (68.29%)	
Hypertension [*N* (%)]				<0.001
No	8,950 (64.06%)	8,071 (67.52%)	879 (43.56%)	
Yes	5,021 (35.94%)	3,882 (32.48%)	1,139 (56.44%)	
Diabetes [*N* (%)]				<0.001
No	11,386 (81.50%)	10,114 (84.61%)	1,272 (63.03%)	
Yes	2,585 (18.50%)	1839 (15.39%)	746(36.97%)	
CVD [*N* (%)]				<0.001
No	12,831 (91.84%)	11,169 (93.44%)	1,662 (82.36%)	
Yes	1,140 (8.16%)	784 (6.56%)	356 (17.64%)	
Education level (%)				<0.001
Less than high school	3,356 (24.02%)	2,744 (22.96%)	612 (30.33%)	
High school	3,109 (22.25%)	2,636 (22.05%)	473 (23.44%)	
More than high school	7,506 (53.73%)	6,573 (54.99%)	933 (46.23%)	

Normally distributed data are presented as the mean ± standard deviation; nonnormal distributed data are presented as median (Q1-Q3) and categorical data are presented using number (percentage). *p* < 0.05 was considered to be statistically significant.

CI, confidence interval; BMI, body mass index; PIR, poverty income ratio; HDL-C, high density lipoprotein cholesterol; LDL-C, low density lipoprotein cholesterol; TC, total cholesterol; TG, triglyceride; ALT, alanine aminotransferase; AST, Aspartate Aminotransferase; FPG, fasting plasma glucose; HbA1c, glycated hemoglobin; BUN, blood urea nitrogen; UA, uric acid; Cr, creatinine; eGFR, estimated glomerular filtration rate; HRR, hemoglobin to red blood cell distribution width ratio; CVD, cardiovascular disease.

Compared with subjects without albuminuria, subjects in the albuminuria group were older and had higher BMI, TG, AST, FBG, HbA1c, BUN, UA, CR, instead, the albuminuria group had lower PIR, LDL-C, TC, eGFR, and HRR. In addition, adults with albuminuria tend to smoke, drink alcohol, have a low level of education, and have a higher prevalence of hypertension, diabetes mellitus, and CVD. However, there was no significant difference in gender, ALT, and HDL-C groups (*p* > 0.05).

### Factors associated with albuminuria

Table 2 describes the results of the univariate analysis. For the unadjusted model, HRR was significantly negatively correlated with albuminuria (*p* < 0.001). In addition, other variables significantly associated with albuminuria were age, BMI, PIR, LDL-C, TC, TG, AST, FBG, HbA1c, BUN, UA, CR, eGFR, smoking, alcohol consumption, hypertension, diabetes mellitus, CVD, and education level (*p* < 0.05).

**Table 2:** Univariate analysis.

Variables	OR	(95%CI)	*p* value
Age (years)	1.04	(1.03, 1.04)	<0.0001
BMI (kg/m^2^)	1.01	(1.00,1.02)	0.0009
PIR	0.91	(0.88, 0.93)	<0.0001
HDL-C (mg/dL)	1.00	(1.00, 1.00)	0.1993
LDL-C (mmol/L)	0.71	(0.66, 0.76)	<0.0001
TC (mmol/l)	0.72	(0.70, 0.75)	<0.0001
TG (mg/dL)	1.00	(1.00, 1.00)	<0.0001
ALT (u/L)	1.00	(1.00, 1.00)	0.1329
AST (u/L)	1.00	(1.00, 1.01)	<0.0001
FBG (mmol/L)	1.19	(1.17, 1.21)	<0.0001
HbA1c(%)	1.47	(1.42, 1.53)	<0.0001
BUN (mg/dL)	1.08	(1.07, 1.09)	<0.0001
UA (mg/dL)	1.19	(1.16, 1.23)	<0.001
CR (mg/dL)	3.92	(3.35, 4.59)	<0.0001
eGFR (mL/min/1.73 m^2^)	0.98	(0.98, 0.98)	<0.0001
HRR (per 0.1 unit)	0.86	(0.84, 0.89)	<0.0001
Sex			
Male	Reference		
Female	0.98	(0.89, 1.07)	0.6347
Race			
Mexican American	Reference		
Other Hispanic	0.92	(0.76, 1.12)	0.4155
Non-Hispanic White	1.06	(0.92,1.22)	0.4077
Non-Hispanic Black	1.09	(0.93, 1.27)	0.2992
Other race	0.79	(0.65, 0.97)	0.0215
Smoking			
Yes	Reference		
No	0.80	(0.73, 0.88)	<0.0001
Drinking			
Yes	Reference		
No	1.26	(1.13, 1.39)	<0.0001
Hypertension			
Yes	Reference		
No	0.37	(0.34, 0.41)	<0.0001
Diabetes			
Yes	Reference		
No	0.31	(0.28, 0.34)	<0.0001
CVD			
Yes	Reference		
No	0.33	(0.29, 0.38)	<0.0001
Education level			
Less than high school	Reference		
High school	0.80	(0.71, 0.92)	0.0012
More than high school	0.64	(0.57, 0.71)	<0.0001

CI, confidence interval; BMI, body mass index; PIR, poverty income ratio; HDL-C, high density lipoprotein cholesterol; LDL-C, low density lipoprotein cholesterol; TC, total cholesterol; TG, triglyceride; ALT, alanine aminotransferase; AST, Aspartate Aminotransferase; FPG, fasting plasma glucose; HbA1c, glycated hemoglobin; BUN, blood urea nitrogen; UA, uric acid; Cr, creatinine; eGFR, estimated glomerular filtration rate; HRR, hemoglobin to red blood cell distribution width ratio; CVD, cardiovascular disease. *p* < 0.05 was considered to be statistically significant.

### Independent correlation between HRR and albuminuria by multivariate piecewise linear regression

**Figure 2:**
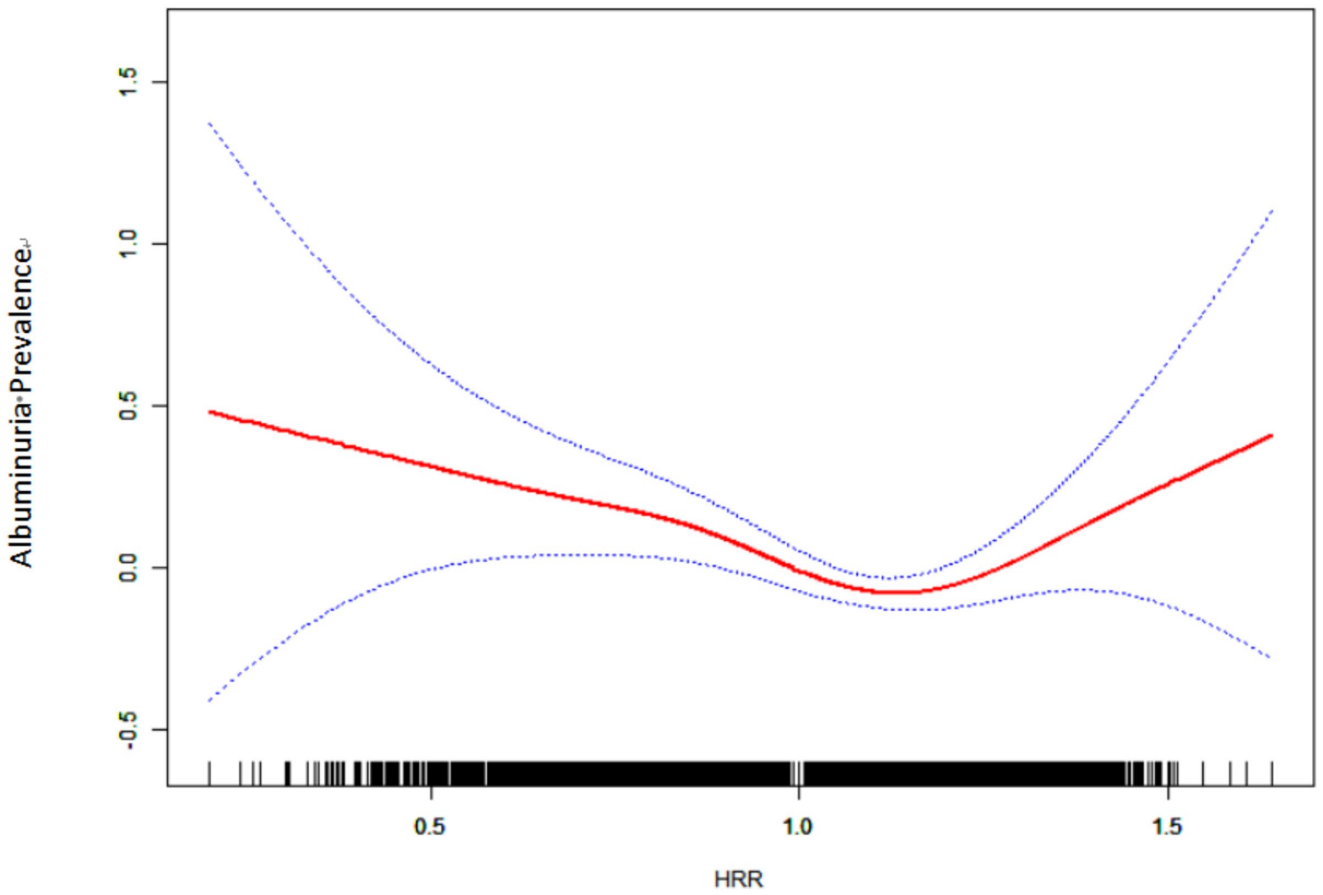
Relationship between HRR and albuminuria by smooth curve fitting. Red band represents the smooth curve fit between variables. Blue bands represent the 95% confidence interval from the fit. Adjustment variables: sex, age, BMI, PIR; TC; TG; ALT; AST; BUN; UA; Cr; eGFR; Race; Smoking; Drinking; Hypertension; Diabetes; CVD; Education level. CI, confidence interval; BMI, body mass index; PIR, poverty income ratio; HDL-C, high density lipoprotein cholesterol; LDL-C, low density lipoprotein cholesterol; TC, total cholesterol; TG, triglyceride; ALT, alanine aminotransferase; AST, Aspartate Aminotransferase; FPG, fasting plasma glucose; HbA1c, glycated hemoglobin; BUN, blood urea nitrogen; UA, uric acid; Cr, creatinine; eGFR, estimated glomerular filtration rate; HRR, hemoglobin to red blood cell distribution width ratio; CVD, cardiovascular disease. *p* < 0.05 was considered to be statistically significant.

After adjusting for gender, age, BMI, PIR, TC, TG, ALT, AST, BUN, UA, CR, eGFR, ethnicity, smoking, drinking status, hypertension, diabetes, CVD, and education level, a smooth curve fit showed a nonlinear relationship between HRR and albuminuria (shown in Figure 2). The inflection point of the HRR was then determined by threshold effects analysis, and the data indicated an inflection point of 1.213 for the HRR. When HRR < 1.213, each 0.1 unit increase in HRR was associated with a 7% reduction in the risk of albuminuria (OR = 0.93, 95% CI 0.90, 0.97; *p* = 0.0007), and conversely, when HRR ≥ 1.213, each 0.1 unit increase in HRR was associated with a 24% increase in the risk of albuminuria (OR = 1.24, 95% CI 1.08, 1.43; *p* < 0.0001) (as shown in Table 3).

**Table 3:** Independent correlation between HRR and albuminuria by multivariate piecewise linear regression.

Inflection point of HRR	OR	95% CI	*p value*
<1.213 (per 0.1 unit)	0.93	(0.90, 0.97)	0.0007
≥1.213 (per 0.1 unit)	1.24	(1.08, 1.43)	0.0031
Log-likelihood ratio			<0.001

Adjustment variables: sex, age, BMI, PIR; TC; TG; ALT; AST; BUN; UA; Cr; eGFR; Race; Smoking; Drinking; Hypertension; Diabetes; CVD; Education level.

CI, confidence interval; BMI, body mass index; PIR, poverty income ratio; HDL-C, high density lipoprotein cholesterol; LDL-C, low density lipoprotein cholesterol; TC, total cholesterol; TG, triglyceride; ALT, alanine aminotransferase; AST, Aspartate Aminotransferase; FPG, fasting plasma glucose; HbA1c, glycated hemoglobin; BUN, blood urea nitrogen; UA, uric acid; Cr, creatinine; eGFR, estimated glomerular filtration rate; HRR, hemoglobin to red blood cell distribution width ratio; CVD, cardiovascular disease. *p* < 0.05 was considered to be statistically significant.

### Subgroup analysis

To assess the consistency of the correlation between HRR and albuminuria. We performed subgroup analyses and interaction tests based on age, gender, race, BMI, diabetes, hypertension, CVD, eGFR, and smoking and drinking status (as shown in Table 4), and the results showed consistent results for this association (interaction *p* > 0.05).

**Table 4:** Subgroup analysis and interaction tests of HRR and albuminuria.

Characteristic	OR (95%CI)	*p*-value	p for interaction
Stratified by age (years)			0.9401
≥60	0.96 (0.91, 1.02)	0.1930	
60	0.98 (0.93,1.03)	0.3956	
Stratified by gender			0.3094
Male	0.99 (0.94, 1.05)	0.7247	
Female	0.95 (0.91, 1.00)	0.0631	
Stratified by race			0.6976
Mexican American	0.93 (0.84, 1.02)	0.1222	
Other Hispanic	0.96 (0.85, 1.08)	0.4800	
Non-Hispanic White	0.99 (0.93, 1.04)	0.6253	
Non-Hispanic Black	0.95 (0.89, 1.02)	0.1923	
Other race	0.92 (0.81, 1.04)	0.1631	
Stratified by BMI			0.5572
≥25	0.96 (0.92, 1.07)	0.0498	
<25	1.00 (0.93, 1.07)	0.9101	
Stratified by diabetes			0.2462
No	0.97 (0.93, 1.02)	0.2065	
Yes	0.93 (0.87, 0.99)	0.0209	
Stratified by hypertension			0.4538
No	0.99 (0.94, 1.05)	0.8196	
Yes	0.95 (0.90, 0.99)	0.0297	
Stratified by CVD			0.7719
No	0.96 (0.92, 1.00)	0.0353	
Yes	0.98 (0.90, 1.08)	0.7467	
Stratified by eGFR			0.8524
≥60	0.97 (0.93, 1.01)	0.1593	
<60	0.96 (0.88, 1.05)	0.3969	
Stratified by smoking			0.1727
No	0.94 (0.90, 0.99)	0.0221	
Yes	0.99 (0.94, 1.04)	0.6630	
Stratified by drinking			0.9570
No	0.94 (0.88, 1.01)	0.0785	
Yes	0.97 (0.93, 1.01)	0.1307	

Adjustment variables: sex, age, BMI, PIR; TC; TG; ALT; AST; BUN; UA; Cr; eGFR; Race; Smoking; Drinking; Hypertension; Diabetes; CVD; Education level.

CI, confidence interval; BMI, body mass index; PIR, poverty income ratio; HDL-C, high density lipoprotein cholesterol; LDL-C, low density lipoprotein cholesterol; TC, total cholesterol; TG, triglyceride; ALT, alanine aminotransferase; AST, Aspartate Aminotransferase; FPG, fasting plasma glucose; HbA1c‌, glycated hemoglobin; BUN, blood urea nitrogen; UA, uric acid; Cr, creatinine; eGFR, estimated glomerular filtration rate; HRR, hemoglobin to red blood cell distribution width ratio; CVD, cardiovascular disease. *p* < 0.05 was considered to be statistically significant.

## Discussion

In this cross-sectional study, we observed a negative correlation between HRR and albuminuria in American adults, which remained stable in fully adjusted models. Smooth curve fitting suggested a nonlinear relationship between HRR and albuminuria with a turning point at 1.213.

It is well known that albuminuria is closely associated with the development of diabetic nephropathy and end-stage renal disease (5). Our study found that albumin decreased with increasing HRR when the HRR was below 1.213. This is similar to the findings of the previous studies. A cross-sectional study conducted by Lin Ning et al. has demonstrated a negative linear association between HRR and CKD, with higher HRR levels indicating a lower prevalence of CKD (22). To investigate the relationship between complete blood count parameters and the incidence of acute kidney injury (AKI) and mortality in patients with Hemophagocytic lymphohistiocytosis (HLH), 585 adult HLH patients were enrolled in Sichuan, China, and a logistic regression model of AKI and 28-day mortality was developed. The multifactorial logistic regression model showed that low HRR (HRR < 0.49) was an independent risk factor for AKI (OR = 1.692) (26). In addition, a retrospective study of 198 patients with renal cell carcinoma (RCC) suggests that low HRR may be considered a new indicator of shorter survival in patients with RCC (27). Since HRR calculation is based on hemoglobin and red RDW, its decrease may be due to low HGB value, high RDW value or both. Previous studies have shown that hemoglobin (every 1 g/dL increase) is negatively correlated with albuminuria (OR 0.92; 95%CI 0.87–0.97) (20). At the same time, elevated RDW is associated with impaired microcirculation function and further increases the risk of albuminuria (28). The possible mechanisms are as follows: First, low HRR levels usually reflect the potential state of chronic inflammation, which plays a key role in the occurrence of albuminuria (8, 29–31). Chronic inflammation inhibits erythropoiesis, leading to decreased hemoglobin, while increasing RDW by disrupting red blood cell maturation and promoting anisocytosis. This imbalance not only indicates impaired hematopoietic function, but also relates to microcirculatory energy impairment (32). Secondly, low HRR levels may indicate impaired oxygen-carrying capacity, which may lead to tissue hypoxia and promote the occurrence of albuminuria (33).

In this study, we used the smooth curve fitting method to describe the nonlinear association and saturation phenomenon between HRR and albuminuria. The saturation effect value of 1.213 may have physiological significance in the relationship between HRR and albuminuria. When HRR is lower than this threshold, HRR may play a stronger protective role in the occurrence of albuminuria; when HRR exceeds this threshold, HRR is positively correlated with the occurrence of albuminuria. The interpretation of this finding needs to be combined with the pathophysiological basis, common influencing factors and interactions of hemoglobin and RDW. Low level HRR is often dominated by chronic inflammation and oxidative stress, which further leads to systemic vascular endothelial dysfunction, glomerular filtration barrier damage, and increased urinary albumin excretion rate (34, 35). However, high levels of HRR often represent a state of high blood viscosity or excessive erythrocyte production, which increases glomerular pressure and accelerates the formation of albuminuria (36). Previous studies have focused on the negative association between HRR and diseases such as cancer, cardiovascular and cerebrovascular diseases and nephropathy (9, 10, 12, 22). The current study lacks evidence that albuminuria increases with HRR. To our knowledge, only one retrospective study of nasopharyngeal carcinoma patients has reported a correlation between higher HRR and poorer prognosis. In the low HRR group, overall survival (OS) and disease-free survival (DFS) were 44.4 months (95% CI: 4.9–83.8) and 15.7 months (95% CI: 0.1–36.2), respectively, but not attainable in the high HRR group (*p* < 0.001), Multivariate analysis showed that low HRR was an independent factor of OS (*p* = 0.004, HR = 3.07, 95% CI:1.444–6.529) and DFS (p < 0.001, HR = 3.94, 95% CI:1.883–8.244). A possible explanation for this is that the study focused on cancer patients receiving chemotherapy and radiotherapy, whose hemoglobin levels varied to varying degrees (37). In addition, a cross-sectional study of 8,868 people found a U-shaped correlation between hemoglobin levels and albuminuria, with an inflection point of 15.5 g/dL, and both high and low levels of hemoglobin were associated with the occurrence of albuminuria (20). It is important to note that although we observed a nonlinear association and saturation between HRR and albuminuria, further studies are needed to determine the optimal level of HRR.

In conclusion, HRR can comprehensively reflect the multifaceted changes in blood status. When anemia is accompanied by inconsistencies in red blood cell production, HRR can more sensitively reflect the pathological status of the patient, which may indicate a wider range of hematological abnormalities and metabolic disorders. In addition, HRR detection is simple to operate and low cost, making it highly accessible and valuable in resource-limited environments. Therefore, HRR provides a new perspective for the early screening of albuminuria.

Subgroup analysis showed that the negative correlation between HRR and albuminuria was more obvious in non-smokers, which may be because smoking itself can lead to various changes in blood status. Previous studies have shown that the red blood cell count and hemoglobin level of patients with smoking showed an increasing trend, which weakened the correlation between HRR and albuminuria (38, 39). It is well known that obesity, hypertension, and diabetes are often accompanied by chronic low-grade systemic inflammation. Inflammatory mediators can lead to red blood cell destruction and suppress erythropoiesis, increasing RDW and exacerbating oxygen transport abnormalities (40–42). Therefore, these populations should pay attention to HRR levels. However, we did not observe similar phenomena in patients with CVD, which requires further research to clarify the exact mechanisms and clinical significance in CVD populations.

### Study strengths and limitations

This study has the following advantages. First, the data were obtained from the NHANES database, which has a large sample size. We included various covariates to adjust for potential confounding factors to enhance the reliability of the results. Second, UACR is an accurate and sensitive indicator with fewer influencing factors and is relatively stable in individuals, allowing for a better assessment of early kidney damage. Finally, through smooth curve fitting and multiple piecewise linear regression model, this study found that there was a nonlinear relationship between HRR and albuminuria.

However, this study has some limitations. First, because of the cross-sectional study design, we were unable to determine causality. Secondly, the subjects of this study were adults aged 20 or older in the United States, and the results could not be generalized to people outside the NHANES sample. Third, although we have adjusted for a variety of confounding factors, there may still be unconsidered confounding factors, such as inflammatory markers and dietary differences, that may affect the conclusions. More prospective studies and clinical trials will be needed to confirm our findings and explore potential mechanisms of action.

## Conclusion

There was a nonlinear correlation between HRR and albuminuria in adults aged 20 or older in the United States. Further prospective research and clinical trials are necessary to confirm our findings and potential mechanisms.

## Data Availability

The datasets presented in this study can be found in online repositories. The names of the repository/repositories and accession number(s) can be found in the article/supplementary material. Publicly available datasets were analyzed in this study. This data can be found here: www.cdc.gov/nchs/nhanes.

## Glossary

CI: confidence interval
BMI: body mass index
PIR: poverty income ratio
HDL-C: high density lipoprotein cholesterol
LDL-C: low density lipoprotein cholesterol
TC: total cholesterol
TG: triglyceride
ALT: alanine aminotransferase
AST: Aspartate Aminotransferase
FPG: fasting plasma glucose
HbA1c‌: glycated hemoglobin
BUN: blood urea nitrogen
UA: uric acid
Cr: creatinine
eGFR: estimated glomerular filtration rate
HRR: hemoglobin to red blood cell distribution width ratio
CVD: cardiovascular disease
CBC: complete blood cell count
RBC: red blood cells
CKD: chronic kidney disease
RDW: cell distribution width
NHANES: National Health and Nutrition Examination Survey of the National Center for Health Statistics
NCHS: National Center for Health Statistics
ACR: albumin creatinine ratio
AKI: acute kidney injury
HLH: hemophagocytic lymphohistiocytosis
RCC: renal cell carcinoma
ROS: reactive oxygen species
OS: overall survival
DFS: disease-free survival
COPD: chronic obstructive pulmonary disease
